# Differences in Health-Related Physical Fitness and Academic School Performance in Male Middle-School Students in Qatar: A Preliminary Study

**DOI:** 10.3389/fpsyg.2022.791337

**Published:** 2022-03-22

**Authors:** Souhail Hermassi, Lawrence D. Hayes, Nilihan E. M. Sanal-Hayes, René Schwesig

**Affiliations:** ^1^Physical Education Department, College of Education, Qatar University, Doha, Qatar; ^2^Institute of Clinical Exercise and Health Science, School of Health and Life Sciences, University of the West of Scotland, Glasgow, United Kingdom; ^3^Department of Orthopaedic and Trauma Surgery, Martin-Luther-University Halle-Wittenberg, Halle, Germany

**Keywords:** anthropometrics, GPA, schoolchildren, performance diagnostic, interactions

## Abstract

This study examined the differences in the level of physical fitness and academic performance among male middle-school children based on different body status categories. A total of 69 male children [age: 12.4 ± 0.7 years; body mass: 58.5 ± 7.2 kg; height: 1.62 ± 0.09 m; and body mass index (BMI): 22.4 ± 3.3 kg/m^2^] participated and were divided into BMI age-adjusted groups (i.e., lowest, middle, and highest BMI). Height, mass, BMI, stork test of static balance, 10 and 15 m sprint as an indicator for speed, hand-grip strength test, agility T-half test, medicine ball throw (MBT), and the Yo-Yo Intermittent Recovery Test level 1 (Yo-Yo IR1) were assessed. School records were retrieved for grade point averages (GPA) of mathematics, science, and Arabic. We found significant group differences regarding anthropometric (height: $\eta_{\text{p}}^{2}$ = 0.24, mass: $\eta_{\text{p}}^{2}$ = 0.33, and BMI: $\eta_{\text{p}}^{2}$ = 0.66), physical (sprint 10 m: $\eta_{\text{p}}^{2}$ = 0.26), and academic (mathematics: $\eta_{\text{p}}^{2}$ = 0.19 and science: $\eta_{\text{p}}^{2}$ = 0.15) performance parameters. The largest difference (*p* < 0.001) was observed between the lowest and highest group for the 10 m sprint. All pairwise differences were between the lowest and highest BMI group or the lowest and middle BMI group. No relevant (*r* > 0.5) correlation between parameters of different dimensions (e.g., anthropometric vs. physical performance parameters) was found. In conclusion, the highest BMI group exhibited similar physical and academic performances than the lowest group. Thus, these data emphasize the importance and appropriateness to engage young Qatari schoolchildren in physical activity as it associates with superior academic performance.

## Introduction

A worldwide epidemiological transition has occurred in the past 20 years, involving a shift from communicable diseases to non-communicable diseases (NCDs), such as obesity and type II diabetes mellitus (Popkin and Doak, 1998; GBD 2015 Risk Factors Collaborators, 2016). In this context, the WHO reported that since 1975, obesity has nearly tripled worldwide (NCD Risk Factor Collaboration (NCD-RisC)., 2017). Being overweight and obese evaluated using body fat is associated with excessive adipose tissue. In contrast, athletes who are extremely muscular show usually a normal or lower body fat but a higher body mass index (BMI) level than average (Longo et al., 2019). Excessive adipose tissue is implicated in several pathologies (Malecka-Tendera and Mazur, 2006; Hermassi et al., 2021a). Concerningly, the obesity epidemic has spread from almost exclusively adult obesity to a greater prevalence of childhood and adolescent obesity in recent decades (Hales et al., 2015).

Physical activity (PA) and exercise improve cardiorespiratory and musculoskeletal fitness, which are associated with increased academic achievement in children and adolescents (Ruiz et al., 2010; Raine et al., 2013; Rauner et al., 2013; El-Sayes et al., 2019; Padulo et al., 2019, p. 13). Specifically, physical fitness (i.e., aerobic fitness, muscular strength, and speed agility) is considered as a powerful, robust measure of health in children and adolescents (Raghuveer et al., 2020). Previous literature is aerobic fitness-centric in relation to academic achievement, concluding higher aerobic fitness is associated with a superior academic achievement. The role of aerobic fitness over muscular strength and speed on driving associations with academic achievement in overweight/obese children is well-described (Cadenas-Sanchez et al., 2020). However, components, such as muscular strength or speed, are less investigated, and there have been calls for further research in this area (Kao et al., 2017).

Previously considered a “disease of affluence” [although this paradigm has attracted some criticism (Ezzati et al., 2005)], obesity now affects those of low-income and middle-income countries and, consequently, displays an inverted U-shaped curve in terms of normalized income and BMI (Ezzati et al., 2005; Sahoo et al., 2015). Socioeconomic status (SES) can thus be considered a covariate; however, cultural experiences of specific countries should also be considered (Dinsa et al., 2012).

Several studies have suggested a positive association between markers of physical health and academic achievement in schoolchildren (Novello et al., 1992; Ruff et al., 2019). Moreover, a systematic review noted that academic performance was improved by some health programs embedded within schools (Murray et al., 2007). Relationships between body mass and brain function (particularly, cognitive function) have been demonstrated in several reviews (Reinert et al., 2013; Martin et al., 2018; Barbosa et al., 2020). Childhood overweightness impacts self-esteem, impairing cognitive and social development (Tremblay et al., 2000; Datar et al., 2004; Hesketh et al., 2004). In addition to evident associations, a systematic review and meta-analysis of 20 studies found that overweight and obese individuals who underwent body mass loss improved their performance across various cognitive domains (Veronese et al., 2017).

The overweight and obesity prevalence among schoolchildren in Qatar is greater (Al-Thani et al., 2018) than the global prevalence of obesity and overweightness (18%) reported by the WHO in 2016. In fact, overweightness and obesity prevalence was 45 and 40% among male and female children and 46 and 41% among Qatari and non-qatari school students, respectively (Al-Thani et al., 2018). Odds of obesity and overweight status were significantly higher among 10–14 and 15–19 years age groups than 5- to 9-year-olds. By sex, male subjects had 1.5 times greater odds of being obese than female subjects, and Qatari nationals had 1.4 times greater odds of obesity than non-qataris (Al-Thani et al., 2018). Due to an often-sedentary lifestyle, the number of overweight adolescents in Qatar is increasing, while PA levels and motor skills are declining.

The association between BMI and academic performance has been reported in several cultures and geographical locations. However, recent systematic reviews found that the relationship between BMI and academic achievement was stronger in American and European samples than in Asian samples, including the Gulf region (Martin et al., 2014). For example, a review of nine studies performed in the United States, Western Europe, South America, and Asia suggested a consistent and significant association (Taras and Potts-Datema, 2005). Similarly, another review showed positive associations between parameters of health (e.g., school-based physical activities) and academic outcomes/performance (Trudeau and Shephard, 2008). Conversely, a systematic review from Santana et al. (2017) reported that in 34 studies, less than half demonstrated an association between obesity and academic performance when controlling for covariates, such as SES and parental education.

Evidence for improved cognitive function as a result of physical health is relatively strong (Keeley and Fox, 2009; Donnelly et al., 2013; Mandolesi et al., 2018). This supposition has led researchers and policymakers to consider the importance of appropriate levels of physical health (including limiting adiposity) for academic performance in children. This is important, as academic performance is a predictor of lifelong health and quality of life across various cultures (Schoenbaum and Waidmann, 1997). Clear mechanistic evidence exists through which physical health leads to improved cognitive health [e.g., cerebral blood flow, brain-derived neurotrophic factor (BDNF), and reduced neurodegeneration, for a detailed review see (Khan and Hillman, 2014)]. However, mechanisms by which academic performance improves lifelong health and quality of life (i.e., the reverse causal relationship) are currently not well-understood. It is widely considered, however, that education, health, and social outcomes are closely interdependent (Kolbe, 2002).

Recent research has begun to establish an association between physical fitness, academic skills, and cognitive variables, demonstrating that PA is not merely coincidentally related to cognitive function (Donnelly et al., 2013; Fernandes et al., 2018; Cadenas-Sanchez et al., 2020; Gil-Espinosa et al., 2020). Aerobic exercise increases levels of neurotrophic factors and brain neuroplasticity, so physical fitness and PA are consequently associated with neurodevelopment and cognitive development (Berchicci et al., 2015). As such, improvements in cognition are seen following aerobic exercise training (Stillman et al., 2016) resultant from signaling-mediated molecular and cellular events resulting in improved cognition (Stillman et al., 2016). Furthermore, increases in gray matter volume and neural activity are apparent (Stillman et al., 2016). Several studies have consistently shown a positive association between fitness level and motor skill acquisition (Etnier and Landers, 1998; Kelsey et al., 2014; Wang et al., 2016). Although it has not been studied directly, improvements in motor performance are potentially mediated by the same mechanisms mediating cognitive improvements.

The negative effects of obesity on academic performance have been demonstrated by some (Esmaeilzadeh and Ebadollahzadeh, 2012; Esmaeilzadeh and Kalantari, 2013; Esteban-Cornejo et al., 2014; Hermassi et al., 2021b) but not all (Santana et al., 2017). However, the interdependence of body mass, physical fitness, and body composition on academic performance in schoolchildren has been examined with academic success associated with higher fitness levels (Datar and Sturm, 2006; Torrijos-Niño et al., 2014).

This cross-sectional study investigated school performance (e.g., Arabic, mathematics, and science) and physical fitness of normal, overweight, and obese middle-school children in Qatar. The primary aim was to identify physical fitness and academic attainment in school students stratified based on BMI-determined obesity. A secondary aim was to examine interactions between physical fitness, BMI, and academic attainment. We hypothesized *a priori* that differences in physical fitness-related health and academic school achievement would be apparent between obese and non-obese school students. In addition, we hypothesized an association between fitness and academic performance would be present.

## Materials and Methods

### Participants

This cross-sectional study followed the guidelines and the Declaration of Helsinki, and the protocol was approved by the university's institutional review board (QU-IRB 1542-FBA/21) and the Ministry of Education and Higher Education Qatar (REF: 18/2021). Independent from the generally accepted criteria for obesity and overweight and population levels, participants were divided into three groups depending on BMI age-adjusted values (Harrington et al., 2013; Hermassi et al., 2021a), namely, lowest (*n* = 21; BMI: 18.0–20.9 kg/m^2^), middle (*n* = 23; BMI: 21.0–22.9 kg/m^2^), and highest (*n* = 25, BMI: > 23.0 kg/m^2^). A total of 69 male schoolchildren (age: 12.4 ± 0.7 years of age, range: 11–14 years; body mass: 58.5 ± 7.2 kg; stature: 1.62 ± 0.09 m; and BMI: 22.4 ± 3.3 kg/m^2^) were analyzed. Of them, 85% (*n* = 59) of the children aged 12 or 13 years. Only 6 (9%) and 4 (6%) were younger (i.e., 11 years) or older (i.e., 14 years). Children were recruited by convenience sampling from one school in a Doha Community (Qatar). Participants were excluded if they were: (1) diagnosed with a psychological disorder (e.g., anxiety, depression, or attention-deficit disorder) or (2) on medication (e.g., antidepressants or medication affecting the nervous system). Moreover, participants that failed to present a signed informed consent form by their legal guardians were further excluded. Study objectives were communicated to the parents, school, and the management team. Parents or legal guardians of the participants were provided with an information sheet explaining the purpose of the experiment and a consent form. Of these participants, 65% returned a signed consent form and were eligible to participate. Participants and their respective guardians and teachers were debriefed about the experimental procedure, right to withdraw from the experiment, and provided with a consent form to complete for participation in the experiment, in accordance with the Helsinki Declaration.

### Procedures and Evaluations

Data collection took place between 08:00 h and 10:00 h every day in an indoor sport court with consistent environmental conditions (temperature 24.5°C ± 0.5°C and relative humidity 65% ± 5%). Participants were asked to maintain regular food and drinks habits but to abstain from drinking caffeinated products, performing vigorous PA, and eating for 24, 4, and 2 h before testing, respectively. Testing was performed in a fixed order over a period of 4 days to ensure consistent fatigue levels and learning effects between participants. The tests for this experiment were selected based on the suitability for middle-school students and to evaluate these dimensions:

1. Sprinting performance (10 and 15 m) and agility performance,
2. Medicine ball overhead throw and hand-grip strength,
3. Endurance performance (Yo-Yo IR1),
4. Static balance performance.

After a general warm-up consisting of 5 min low-intensity running, 3 × 15 m progressive accelerations and a maximal 20 m sprint, interspersed with 3 min periods of passive recovery were performed. In addition to the low-intensity running, submaximal dynamic stretches and throws were performed, consistent with our previous work. Day 1 consisted of anthropometric testing and the Yo-Yo IR1. On day 2, the stork test of static balance was performed. Day 3 consisted of sprint performance and handgrip force. On day 4, testing consisted of the agility T-half test and medicine ball throw. Test-retest reliability was assessed by repeating fitness tests from day 1 to 3 after 2 weeks of the initial testing period. The second set of test scores generated for the test-retest reliability purposes was included in final analyses. Anthropometric and Yo-Yo IR1 test assessments were performed once at the initial testing period, for players' limited time commitments and schedules.

## Day 1

### Anthropometry

Anthropometric measurements incorporated body height (Holtain stadiometer, Crosswell, Crymych, Pembrokeshire, United Kingdom) and body mass (model TBF 105; Tanita Corporation of America, Inc, Arlington Heights, Illinois) measured to the nearest 0.1 cm and 0.1 kg. BMI was calculated as the ratio between body mass (kg) and body height squared (m^2^).

### The Yo-Yo Intermittent Recovery Test Level 1

The Yo-Yo IR1 was conducted in line with Krustrup et al. (2003). A standardized warm-up consisted of 5 min of low-intensity running. Next, 20 m shuttle runs were performed at increasing velocities until exhaustion, with 10 s intervals of active recovery (2 × 5 m of jogging) between runs. The test was terminated if it met objective criteria (with the participant twice failing to reach the front line in time) and/or subjective criteria (with the participant feeling unable to complete another shuttle at the required speed). The total distance covered was considered as the test “score.”

## Day 2

### Static Balance Performance

To assess static balance, we utilized the stork balance test (Miller, 2002). Participants stood with their opposite foot against the inside of the supporting knee with both hands on the hips. On the “go” signal, subjects raised the heel from the floor and held this position for as long as possible. The test was terminated when the heel of the supporting leg touched the ground, or the foot moved away from the kneecap. The test was timed using a stopwatch.

## Day 3

### Sprint Tests

A 15-min warm-up with 10 min running, change of direction activities, and dynamic stretching was performed. Next, participants sprinted 15 m from a standing position 0.2 m behind the first photocell beam. The 15 and 30 m sprint times were recorded by paired photocells (Racetime 2 SF, Microgate, Italy) located 1 m above the ground at the start and finish. Three trials were separated by 6–8 min of recovery, and the fastest trial was retained for further analyses.

### Handgrip Strength Test

A standard adjustable digital hand-grip dynamometer (T.K.K. 5401, Tokyo, Japan) was employed to measure grip strength of the dominant hand, with a sensitivity of 10 N. The anthropometric apparatus and hand-grip dynamometer were calibrated before use. Participants were tested 3 min after a separate warm-up and before a throwing velocity test, following the anthropometry assessment. This test was conducted with the arm extended parallel to the body. Participants were not allowed to display movements of the arm or wrist. Peak force was recorded. Players performed two repetitions of maximum intensity with 3 min break between each repetition to minimize fatigue. The final analyses included the best trial only.

## Day 4

### Change of Direction (T-Half Test)

Prior to each test, a 10 min warm-up consisting of jogging, jumping, lateral displacements, and dynamic stretching was performed. Electronic timing sensors (photocells, Kit Racetime 2 SF, Microgate, Italy) were employed to record T-half tests (Sassi et al., 2009) data. The electronic timing sensors were set 0.75 m above the floor, 3 m apart, and facing each other at the starting line A. Participants started each trial with their front foot 0.2 m behind line A. Next, participants sprinted forward to cone B and touched its base with their right hand. Without crossing their feet and facing forward, participants shuffled to the left of cone C and touched the base of this cone with their left hand. Thereafter, participants shuffled right to cone D, touched its base with their right hand, then ran back to the left of the cone, and touched its base. At last, participants ran backward, returning to line A as quickly as possible. Participants had to repeat a trial if they crossed one foot in front of another, failed to touch the base of the cone, and/or failed to face forward throughout the trial. Participants repeated until they completed two successful trials; there was a 3 min break between the trials, and only the best trial was included in the final analyses.

### Medicine Ball Throw

Prior to this test, participants performed a 5 min warm-up that included a 3 min run and dynamic activities. Throws were performed using rubber medicine balls with a diameter of 21.5 cm and a weight of 2 kg. Participants were debriefed about the optimal technique, determining an optimal release angle for obtaining the maximum distance (Negrete et al., 2010). The sitting participant clasped the medicine ball using both hands and pushed the ball forcefully from their chest when given a signal. The score was determined based on the place the ball landed from the sitting line's front. Each participant performed three trials with 1 min break between, and the best result was reported to 0.01 m.

### Academic Performance

Academic performance was evaluated through school records. Academic performance consisted of actual grade point average (GPA) and score (0–100) as endorsed in the Qatar State in mathematics and science from the second semester of the academic year 2020–2021. The reason for only including two academic subjects was due to our interest in science-related courses. We focused on mathematics and science due to the greater association with fitness.

### Statistical Analysis

The Statistical Package for the Social Sciences (SPSS) version 28.0 (IBM, Armonk, NY, USA) software was utilized to perform all analyses. Retest reliability was described using the intraclass correlation coefficient (ICC) and the coefficient of variation (CV) (Schrama et al., 2014). Previously reported guidelines helped to inform reliability (Shrout and Fleiss, 1979; Hopkins, 2000; Portney and Watkins, 2009; Hopker et al., 2010). A power calculation (nQuery Advisor 4.0; Statistical Solutions, Saugus, MA, USA) showed with *n* = 7 in each group; we would have been able to detect a mean difference of 0.4 s in 10 m sprint using a two-sided *t*-test with the α level of 0.05 and a pooled standard deviation (SD) of 0.3 s, with a statistical power of 0.8 (Bortz, 1999). Data were analyzed for homogeneity of variance (Levene's test), and normality (Shapiro-Wilk test). Based on the sample size of *n* = 69 and the symmetrical sample distribution (*n* = 21, *n* = 23, and *n* = 25), it was possible and legitimate to use parametric tests (Weiß, 2002), such as one-way analysis of variance (ANOVA), to examine the anthropometric and performance differences between BMI groups. Differences were considered meaningful if *p* < 0.05 and partial eta-squared ($\eta_{\text{p}}^{2}$) > 0.15 (Bortz, 1999; Richardson, 2011), to refrain from overestimating the differences. *T*-tests with Bonferroni correction were conducted for pairwise comparison purposes (0.05/17 or *p* = 0.003) to protect against type I error (Bortz, 1999). Pearson's product moment correlation coefficients (*r*) were conducted to determine relationships between anthropometric, physical performance, and academic performance variables and interpreted employing Cohen's thresholds (Cohen, 1988). Magnitudes of correlations (*r*) were thus interpreted as follows: *r* < 0.1; the correlation was trivial; 0.1–0.3, it was small; in the range of 0.3–0.5, moderate; 0.5–0.7, large; 0.7–0.9, very large, and 0.9–1.0, almost perfect (Cohen, 1988).

## Results

### Intrarater Reliability

All six performance tests showed excellent relative reliability (ICC ≥ 0.75). ICC ranged from 0.87 (stork balance test) to 1.00 (all other tests with the omission of medicine ball throw). Apart from the stork balance test (CV = 13.9%), all variables had excellent absolute reliability, with CV < 5% (Table 1).

**Table 1:** Physical fitness tests obtained from two sessions (time interval: *n* = 23).

**Test**	**Session one mean ±SD**	**Session two mean ±SD**	**ICC (95% CI)**	**CV (%) (95% CI)**
10 m sprint (s)	2.51 ± 0.36	2.53 ± 0.37	**1.00** (0.99–1.00)	**0.6** (0.4–0.9)
15 m sprint (s)	3.15 ± 0.35	3.18 ± 0.35	**1.00** (0.98–1.00)	**0.7** (0.5–1.1)
Agility T-half test (s)	8.40 ± 1.67	8.53 ± 1.70	**1.00** (0.98–1.00)	**1.6** (1.2–2.5)
Medicine ball throw (m)	4.95 ± 0.80	4.80 ± 0.80	**0.97** (0.90–0.99)	**3.7** (2.8–5.8)
Stork balance test (s)	46.1 ± 22.2	42.6 ± 15.0	**0.87** (0.70–0.95)	**13.9** (10.9–23.6)
Handgrip force (N)	29.6 ± 12.3	28.2 ± 12.3	**1.00** (0.78–1.00)	**2.0** (1.5–3.1)

*Descriptive statistics [mean ± standard deviation (SD)] and intrarater reliability are presented for each test. Intraclass correlation coefficient (ICC) ≥ 0.75 and coefficient of variation (CV) ≤ 10% marked in bold*.

### Normal Distribution and Variance Homogeneity

Only two variables (i.e., sprint 15 m: *p* = 0.285; medicine ball throw: *p* = 0.059) were normally distributed. Only the parameters, such as 10 m sprint (*p* = 0.001), Yo-Yo IR1 (*p* = 0.001), and science (*p* = 0.007), were heterogeneous in variance. Otherwise, all *p*-values were >0.110.

### Age and Anthropometric Data

All three groups were not different concerning age ($\eta_{\text{p}}^{2}$ = 0.03) but showed differences regarding anthropometric parameters (Table 2).

**Table 2:** Comparison of age and anthropometric characteristics between three groups.

	**Lowest group (*n* = 21)**	**Middle group (*n* = 23)**	**Highest group (*n* = 25)**	**ANOVA**		**Significant partial effects**
				***p***	** $\eta_{\text{p}}^{2}$ **	***p***
Age (years)	12.6 ± 0.81	12.3 ± 0.70	12.3 ± 0.68	0.344	0.03	–
**Anthropometric parameters**						
Body height (m)	1.68 ± 0.06	1.61 ± 0.09	1.58 ± 0.08	**<0.001**	**0.24**	Lowest vs. middle: 0.012
						Middle vs. highest: <0.001
Body mass (kg)	53.7 ± 5.17	57.3 ± 6.20	63.6 ± 6.47	**<0.001**	**0.33**	Highest vs. middle: 0.002
						Highest vs. lowest: <0.001
BMI (kg/m^2^)	19.1 ± 1.46	22.1 ± 1.90	25.6 ± 2.28	**<0.001**	**0.66**	Lowest vs. middle: <0.001
						Lowest vs. highest: <0.001
						Middle vs. highest: <0.001

*Values are given as mean ± SD. Significant effects (main effect criteria: p < 0.05 and $\eta_{\text{p}}^{2}$ ≥ 0.15) marked in bold*.

The largest difference was observed for the parameter BMI ($\eta_{\text{p}}^{2}$ = 0.69). The BMI of the lowest group (19.1 ± 1.5 kg/m^2^) was markedly lower than for the middle group (25.5 ± 2.2 kg/m^2^) and the highest group (25.6 ± 2.3 kg/m^2^). Differences in body height ($\eta_{\text{p}}^{2}$ = 0.30) and body mass ($\eta_{\text{p}}^{2}$ = 0.35) were of a smaller magnitude.

### Physical Performance Academic Performance Data

Regarding physical performance and academic performance parameters, we found three significant group differences (i.e., 10 m sprint, mathematics, and science) (Table 3). The largest difference was calculated for 10 m sprint ($\eta_{\text{p}}^{2}$ = 0.26).

**Table 3:** Comparison of physical performance and academic characteristics between three groups (differentiation criterion: percent body fat).

	**Lowest group (*n* = 21)**	**Middle group (*n* = 23)**	**Highest group (*n* = 25)**	**ANOVA**		**Significant partial effects**
				***p***	** $\eta_{\text{p}}^{2}$ **	***p***
**Physical performance parameters**						
Yo-Yo IR 1 (m)	480 ± 185	628 ± 281	532 ± 184	0.086	0.07	–
Medicine ball throw (m)	4.96 ± 0.68	4.64 ± 0.67	5.16 ± 0.61	0.027	0.10	Middle vs. highest: 0.023
Agility T-half (s)	8.58 ± 1.07	8.03 ± 1.05	8.66 ± 1.55	0.187	0.05	–
Stork balance test (s)	44.5 ± 22.7	51.6 ± 25.8	43.5 ± 23.0	0.456	0.02	–
Handgrip force (N)	27.4 ± 9.56	26.0 ± 8.28	30.1 ± 11.9	0.357	0.03	–
Sprint 10 m (s)	2.53 ± 0.34	2.25 ± 0.22	2.68 ± 0.34	**<0.001**	**0.26**	Lowest vs. middle: 0.012
						Middle vs. highest: <0.001
Sprint 15 m (s)	3.24 ± 0.38	3.11 ± 0.34	3.25 ± 0.29	0.287	0.04	–
**Academic performance parameters**						
Arabic	77.0 ± 9.31	84.4 ± 9.02	79.7 ± 9.41	0.029	0.10	Lowest vs. middle: 0.028
Mathematics	81.0 ± 8.26	87.3 ± 4.73	80.8 ± 5.79	**0.001**	**0.19**	Lowest vs. middle: 0.005
						Middle vs. highest: 0.002
Science	77.6 ± 10.2	85.0 ± 5.81	79.2 ± 6.86	**0.005**	**0.15**	Lowest vs. middle: 0.007
						Middle vs. highest: 0.035

*Values are given as mean ± SD. Significant effects (main effect criteria: p < 0.05 and $\eta_{\text{p}}^{2}$ ≥ 0.15) marked in bold*.

Significant effects were observed only between the middle and highest groups (i.e., medicine ball throw, sprint 10 m, mathematics, and science) or between lowest and middle participants (i.e., sprint 10 m, Arabic, mathematics, and science). The largest difference (*p* < 0.001) was observed between overweight (2.25 ± 0.22 s) and obese (2.68 ± 0.34 s) subjects for the 10 m sprint. The largest number of pairwise differences (5) was calculated for academic performance parameters. The differences ranged from *p* = 0.002 (mathematics: middle vs. highest) to *p* = 0.035 (science: middle vs. highest).

In contrast to the academic performance (all maxima in the middle group), the performance maxima concerning physical performance are distributed among the three groups (Table 3). Most of the maxima (5) were found for the middle group (parameters: Yo-Yo IR1, sprint 10 m, sprint 15 m, storck balance test, and agility T-half). The subjects of the highest group showed the highest performance for the medicine ball throw (5.16 ± 0.61 m) and handgrip force (30.1 ± 11.9 N).

### Relationships Between Parameters

We did not find any relevant (*r* > 0.5) correlation between parameters of different dimensions (e.g., academic vs. physical performance; anthropometric vs. physical performance). The parameters medicine ball throw and handgrip force showed the highest interaction (*r* = 0.470) with each other. Regarding the differentiating parameter BMI, all product moment correlations were below *r* = 0.303 (95% CI: 0.071–0.504, medicine ball throw).

## Discussion

This study explored physical fitness and academic attainment differences in school students stratified by BMI. An initial outcome was that participants with the lowest BMI did not exhibit the greatest performance in any parameter. The middle group, however, displayed the greatest physical performance level in 5 of 7 parameters (71%), but differences only reached the *p* < 0.05 level for the 10 m sprint. Regarding academic performance, the middle-BMI group exhibited the greatest performance in all three parameters, particularly mathematics and science. There were no relevant relationships between both dimensions (i.e., physical vs. academic performance), and so we surmised no transfer effects were evident. In this context should be noted that a slightly increased BMI is an indication for acceleration in the sense of sexual maturity (i.e., biological maturation), with differences in anthropometrics due to various patterns in the growth spurt (Tanner, 1989). The growth spurt is generally preceded by a quick increase in body fatness, a phenomenon known as the “prepubertal fat wave” (Tanner, 1989). However, a highly significant variability in timing biological maturation can be reported between individuals of the same sex, resulting in early and late maturers (Tanner, 1989; Armstrong and McManus, 2011), which may imply a substantial difference in body mass and fat associated with sprint performance. As information about the biology maturation of the school students is missing, we must consider that some results (e.g., handgrip force and medicine ball throw) can be more a function of biology maturation than body composition alone. This could partly explain why the lowest and highest BMI groups performed worse in several tests. It could be that the highest and lowest BMI group were on different maturation trajectories (i.e., early or late maturers), and each physical fitness attribute peaks at different stages of maturation according to the peak height velocity (Towlson et al., 2021).

### Physical Performance Data

During school physical education classes, short sprints, changes of direction, accelerations, and decelerations are fundamental abilities developed and utilized (Hermassi et al., 2021c). It is well-known that obesity is negatively associated with physical fitness. However, it has been reported that BMI is a poor surrogate for obesity (which is purportedly a disease of excessive fatness, although not if measured by BMI) in adolescents (Shang et al., 2010; Kruschitz et al., 2013; Hermassi et al., 2021d). We found significant group differences regarding 10 m sprint performance, and the largest difference was observed between the middle and highest BMI group (*p* < 0.001). This has implications for school student's success as they commonly sprint for ~10–30 m in game-defining epochs during physical education classes.

Most of the maximal values (5) were exhibited by the middle BMI group (parameters: Yo-Yo IR1, 10 m sprint, 15 m sprint, stock balance test, and agility T-half). The highest BMI group exhibited the greatest performance for the medicine ball throw and handgrip force. This is relatively unsurprising, as the highest BMI group would exhibit the greatest absolute volume of lean mass, which is a key determinant of muscle force (Bandini et al., 1990; Esmaeilzadeh and Ebadollahzadeh, 2012; Esmaeilzadeh and Kalantari, 2013). In this context, Esmaeilzadeh and Kalantari (2013) controlled for body fat mass and observed that in some physical fitness tests (such as sit and reach, standing long jump, and run speed tests), overweightness was associated with improved performance compared with underweight counterparts, indicating fat mass as a limiting factor for test performance. Results of Esmaeilzadeh and Kalantari (2013) indicated that underweight adolescents possessed the highest cardiorespiratory fitness among the weight statuses, but being underweight was related to poorer performance of some physical fitness tests (primarily related to muscle strength). It is possible that their poorer performance resulted from lower fat-free mass compared with that in normal and overweight counterparts, which was supported by these results when controlling for fat-free mass.

### Academic Performance

We found relevant group differences regarding the academic performance parameters mathematics ($\eta_{\text{p}}^{2}$ = 0.19) and science ($\eta_{\text{p}}^{2}$ = 0.15). For Arabic language ($\eta_{\text{p}}^{2}$ = 0.10), the group difference did not reach the level of relevance (*p* < 0.05 and $\eta_{\text{p}}^{2}$ ≥ 0.15). Furthermore, the largest number of pairwise differences (5) was calculated for academic performance parameters. The differences ranged from *p* = 0.002 (mathematics: middle vs. highest) to *p* = 0.035 (science: middle vs. highest). These data corroborate previous cross-sectional studies that observed fitter students exhibited greater academic attainment (Davis and Cooper, 2011; Hraste et al., 2018). To date, some investigations reported an association between body composition (not part of our study) and academic performance (Sabia, 2007; Sigfúsdóttir et al., 2007; Shore et al., 2008). For example, our previous work demonstrated that a nonobese adolescent group had a higher mean academic performance than the obese group (Hermassi et al., 2021d). The observed difference between groups was markedly greater for mathematics ($\eta_{\text{p}}^{2}$ = 0.367) than for science ($\eta_{\text{p}}^{2}$ = 0.195).

It is well-known that adiposity has negative effects on cognition, learning, and memory (Li et al., 2008; Yu et al., 2009). However, weight-related bias or discrimination may further influence self-esteem and behavioral problems, mediating or moderating obese children's academic performance (Fowler-Brown et al., 2010; Griffiths et al., 2010). Uncertainty remains whether excess adiposity itself affects academic performance or if this effect is mediated by other factors observed in individuals with obesity. A systematic review noted a relationship between obesity, cognitive function, and academic attainment in adolescents (Kamijo et al., 2012). In this study, the highest group had poorer academic achievement, suggesting that being obese during adolescence has profound academic consequences. Paradoxically, other studies have observed no effect of obesity on academic achievement in children (Kwak et al., 2009; Van Dusen et al., 2011). Reasons for result divergence remain unclear, but differences in methods for quantifying obesity (i.e., body fat or BMI), participant characteristics, growth stages, or analysis procedures for academic attainment (Kwak et al., 2009; Ruiz et al., 2010) may explicate some ambiguity. Furthermore, if previous studies are interpreting results solely on the alpha level and using an arbitrary threshold of *p* < 0.05 with small sample sizes, it is possible these studies fell afoul of type II error. In this context, based on Cohen's thresholds (Cohen, 1988), the present magnitudes were classified as “small,” and thus the highest BMI group may only experience a small negative effect on academic performance, which may be missed by underpowered research studies. If studies use different analysis procedures for academic attainment, this may also explain differences in results as obesity is not associated with lower test scores (Datar and Sturm, 2006) but does associate with lower GPA (Li et al., 2008). This is puzzling, as one may assume lower test scores would result in lower GPA; however, it does emphasize (a) the importance of specifying how academic performance was quantified and (b) the multifactorial nature of how obesity associates with GPA, as it is clearly not simply a case of poorer test performance.

### Limitations

Limitations of this investigation include not assessing sexual maturity (i.e., biological maturation) as growth spurt timing differences could influence BMI and relationships between BMI and physical fitness. A further limitation is that since the study was cross-sectional, it is difficult to attribute causality. For a wider interpretation and scope, it is necessary to involve female subjects. In addition, the parameter body fat should be assessed to distinguish between fat and lean mass. Finally, we did not use the generally accepted criteria for obesity and overweight. Instead of this, we divided the sample into tertiles based on BMI.

## Conclusion

This study determined physical fitness and academic achievement in BMI-stratified obese and non-obese adolescents. Surprisingly, the middle BMI group (not the lowest group) displayed the highest physical and academic performance levels in most parameters. These data suggest that an excess body mass may have an impact on academic attainment in children. Conversely, students with higher academic performance may be more able to maintain a healthy body mass. Future studies utilizing the gold standard for body composition measures or lean mass measures (e.g., hydrostatic weighing or D_3_-creatine) and additional parameters (e.g., sexual maturation status and SES) are required to confirm our preliminary observations.

## Data Availability Statement

The raw data supporting the conclusions of this article will be made available by the authors, without undue reservation.

## Ethics Statement

The studies involving human participants were reviewed and approved by Qatar University's institutional review board (QU-IRB 1542-FBA/21) and the Ministry of Education and Higher Education Qatar (REF: 18/2021). Written informed consent to participate in this study was provided by the participants' legal guardian/next of kin.

## Author Contributions

SH and RS conceptualized the study and validated the manuscript. SH contributed to methodology, project administration, funding acquisition, visualization, formal analysis, investigation, resources, software, and wrote the original draft preparation. RS contributed to supervision and data curation. SH, NS-H, and LH contributed to writing, reviewing, and editing. All authors have read and agreed to the published version of the manuscript.

## Funding

This work was jointly supported by Qatar University QUCG-CENG-22/23-522 (Collaborative Grant). The findings achieved herein are solely the responsibility of the authors.

## Conflict of Interest

The authors declare that the research was conducted in the absence of any commercial or financial relationships that could be construed as a potential conflict of interest.

## Publisher's Note

All claims expressed in this article are solely those of the authors and do not necessarily represent those of their affiliated organizations, or those of the publisher, the editors and the reviewers. Any product that may be evaluated in this article, or claim that may be made by its manufacturer, is not guaranteed or endorsed by the publisher.
